# Building trust with marginalized communities in participatory acoustic monitoring through dynamic consent

**DOI:** 10.1111/cobi.70222

**Published:** 2026-03-09

**Authors:** Joycelyn Longdon, Emmanuel Acheampong, Jennifer Gabrys, Alan Blackwell, Ben Ossom, Adham Ashton‐Butt

**Affiliations:** ^1^ Computer Laboratory University of Cambridge Cambridge UK; ^2^ Department of Silviculture and Forest Management Kwame Nkrumah University of Science and Technology Kumasi Ghana; ^3^ Department of Sociology University of Cambridge Cambridge UK; ^4^ Department of Wildlife and Range Management Kwame Nkrumah University of Science and Technology Kumasi Ghana; ^5^ British Trust for Ornithology Thetford UK

**Keywords:** citizen science, conservation data justice, ecoacoustics, Indigenous Peoples and local communities, participatory monitoring, passive acoustic monitoring, ciencia ciudadana, comunidades locales y pueblos indígenas, justicia en los datos de conservación, monitoreo acústico pasivo, monitoreo participativo, 被动声学监测, 参与式监测, 土著人民与当地社区, 生态声学, 保护数据正义, 公民科学

## Abstract

There exists a growing suite of technologies that support significant and exciting progress in biodiversity conservation and research. Citizen scientist participation is common in this research and often focuses on data collection and labeling. Yet, ongoing challenges exist concerning trust in participatory monitoring projects engaging Indigenous Peoples or local communities. These challenges are rooted in the proliferation of Western‐centric approaches to engagement and uneven power dynamics between researchers and participants. Using passive acoustic monitoring (PAM) as a model, we explored how researchers can build trust in participatory research with conservation technologies. Working closely with 12 members of a forest fringe community in Ghana, we conducted semistructured interviews investigating community members’ perceptions of and concerns with ecoacoustic technologies and a series of participatory workshops exploring ecoacoustic data practices. Through our interviews, we found that 4 key themes—questioning, agency, proof, and knowledge—shaped community members’ sense of trust when engaging with conservation and technology systems or practices. Our engagements highlighted a need for a dynamic consent process, which entails a set of engagements and activities tailored to community members’ needs, to ensure they could make informed decisions on their involvement in research projects. To facilitate more ethical and just community engagements that result in higher quality data and more successful conservation outcomes, we recommend that researchers working with conservation technologies and marginalized communities respond to suspicion, address agency, center community knowledge, and demonstrate data practices.

## Abstract (es)

Generación de confianza en las comunidades marginadas que participan en el monitoreo acústico por medio del consentimiento dinámico

**Resumen:**

Cada día hay un mayor conjunto de tecnologías que respaldan avances significativos y emocionantes en la conservación y la investigación de la biodiversidad. La participación de científicos ciudadanos es habitual en esta investigación y, a menudo, se centra en la recopilación y el etiquetado de datos. Sin embargo, existen retos continuos en relación con la confianza en los proyectos de seguimiento participativo en los que participan pueblos indígenas o comunidades locales. Estos retos tienen su origen en la proliferación de enfoques centrados en Occidente para la participación y en las dinámicas de poder desiguales entre investigadores y participantes. Utilizamos el monitoreo acústico pasivo (MAP) como modelo para explorar cómo los investigadores pueden generar confianza en la investigación participativa con tecnologías de conservación. En colaboración estrecha con 12 miembros de una comunidad situada en los límites del bosque en Ghana, realizamos entrevistas semiestructuradas para investigar las percepciones y preocupaciones de los miembros de la comunidad con respecto a las tecnologías ecoacústicas, y una serie de talleres participativos para explorar las prácticas de datos ecoacústicos. Con nuestras entrevistas descubrimos que cuatro temas clave —el cuestionamiento, la agencia, la prueba y el conocimiento‐ moldeaban la sensación de confianza de los miembros de la comunidad al participar en sistemas o prácticas de conservación y tecnología. Nuestras interacciones destacaron la necesidad de un proceso de consentimiento dinámico, que implique una serie de compromisos y actividades adaptados a las necesidades de los miembros de la comunidad, para garantizar que puedan tomar decisiones informadas sobre su participación en proyectos de investigación. Para facilitar una participación más ética y justa de la comunidad, que se traduzca en datos de mayor calidad y resultados de conservación más satisfactorios, recomendamos que los investigadores que trabajan con tecnologías de conservación y comunidades marginadas respondan a las sospechas, aborden la agencia, se centren en el conocimiento de la comunidad y demuestren las prácticas de datos.

## 通过动态同意机制在参与式声学监测中建立与边缘化社区的信任

**【摘要】** 一系列支持生物多样性保护与研究取得重大进展的技术正在不断涌现。公民科学家参与此类研究已成常态, 其工作常聚焦于数据采集与标注。然而, 在涉及土著人民或当地社区的参与式监测项目中, 常常存在信任方面的问题。这些挑战的根源在于以西方为中心的参与模式普遍存在, 以及研究者与参与者之间权力动态失衡。本研究以被动声学监测为模型, 探讨了研究者如何利用保护技术建立参与式研究的信任机制。我们与12名生活在加纳森林边缘社区的成员紧密合作, 通过半结构化访谈探究了社区成员对生态声学技术的认知与顾虑, 并举办系列参与式研讨会探索了生态声学的数据实践。我们从访谈中总结了四个核心主题——质疑、能动性、证据, 以及知识, 这构成了社区成员在接触保护和技术系统或实践时的信任基础。我们的实践强调了需要建立动态同意机制, 根据社区成员的需求来设计一系列参与活动, 确保其在参与研究项目的过程中能基于充分知情而做出决策。为促进更符合伦理与公平的社区参与, 获得更高质量的数据和更成功的保护成果, 我们建议使用保护技术且与边缘化社区互动的研究者, 应在保护工作中回应社区疑虑、解决能动性问题、重视社区知识, 并展示数据实践。**【翻译:胡怡思;审校:聂永刚】**

## INTRODUCTION

Aided by falling device hardware costs and increasingly powerful analysis methods, there exists a growing suite of conservation technologies that can support significant and exciting progress in conservation research. From drones that facilitate reforestation to camera traps that monitor wildlife biodiversity, conservation technologies are being used to observe ecosystems across a range of spatial and temporal scales.

Among these technologies, passive acoustic monitoring (PAM) has emerged as an increasingly popular and successful approach and has “revolutionis[ed] faunal survey methods” (Sugai et al., 2019). This technology encompasses the fields of bio‐ and ecoacoustics and involves acoustic sensors “deployed in the field for extended periods of time to estimate species occupancy, abundance and population density, to monitor animal behavior, and to survey and monitor ecological communities” (Browning et al., 2017). PAM has been likened to a “time capsule” (Deichmann et al., 2018) in that it facilitates long‐term storage of field recordings that can be reanalyzed by a variety of researchers in the future. There are many levels at which environmental acoustic recordings can be analyzed (Oliver et al., 2018). For example, at the coarse soundscape level, data can be analyzed using unsupervised machine learning, clustering, and acoustic indices (Napier et al., 2024; Sethi et al., 2020; Towsey et al., 2014), and at the species level (Stowell, 2022), data can be analyzed with supervised machine‐learning techniques (Oliver et al., 2018).

The development and deployment of conservation technologies, such as PAM, are supported by the substantial contributions of citizen science (CS) and participatory monitoring (PM) projects (Chandler et al., 2017; Green et al., 2020; Kobori et al., 2016; Skarlatidou et al., 2024), where volunteers collaborate with scientists to collect and analyze biodiversity data (Chandler et al., 2017; Pocock et al., 2018). In PAM, there is a long tradition of engaging the expertise and labor of hobbyists, such as birders (Jäckel et al., 2021; Loureiro et al., 2019; Sullivan et al., 2014; Wood et al., 2022; Zilli, 2015).

Despite the existing contributions and the immense potential for CS in biodiversity monitoring, there exist ethical, social, and justice implications that must be attended to. A key limitation (especially outside a Western context) is the lack of ability to build and retain trust with participants (Benyei et al., 2023; Chiaravalloti et al., 2022; Eleta et al., 2019; Horowitz, 2010; Saif et al., 2022; Stern & Coleman, 2015). Lack of trust is a key factor perpetuating community reluctance to collaborate and cooperate in conservation initiatives (Saif et al., 2022) and a significant research challenge for conservation practitioners (Thompson et al., 2020).

Conservation has a colonial history that has seen Indigenous and local communities dispossessed from their ancestral or community lands, often through the militarization of conservation (Duffy et al., 2019; Saif et al., 2022). With this violent history perpetuated through ongoing human rights abuses (Saif et al., 2022), there remains an observable and evidenced legacy of distrust of conservation by many in the Global South. Given that “the extraction of value through data represents a new form of resource appropriation on par with…historical colonialism” (Couldry & Mejias, 2023), the distrust in conservation is amplified by neocolonial approaches to technology development and data collection.

### Importance of trust in conservation

There exist many conceptualizations of trust across various fields; however, most definitions highlight the state of vulnerability that trust shifts the trustee into (Horowitz, 2010; Mayer et al., 1995; Stern & Coleman, 2015; Vegt et al., 2023). Trust is intimately connected to community perceptions of justice (Saif et al., 2022), which, although gaining increased attention in conservation discourse, is less evidenced in practice. As it stands, much of this research fails to take an interdisciplinary approach to trust building (Stern & Coleman, 2015) and instead focuses on “securing trust as a means to an end for achieving cooperation for conservation success” (Saif et al., 2022), rendering it a technical practice that is not used to interrogate “the political status quo that may be perpetuating distrust in the first place” (Saif et al., 2022). Although researchers often detail the free, prior, and informed consent (FPIC) obtained from people they work with, there are doubts concerning its effectiveness and ability to enhance equity in conservation (Larsen & Chanthavisouk, 2024). Given the historic and ongoing power dynamics and systems of oppression that operate in forest ecosystems, “the collaboration of local communities with newcomers, such as professional scientists, can exacerbate existing tensions related to indigeneity, ethnicity, and gender, or provoke new ones” (Moustard et al., 2021). Especially where conservation technologies are concerned, monitoring can, knowingly and unknowingly, become a form of surveillance (Adams, 2019; Sandbrook et al., 2021) and thus breach the rights and privacy needs of communities living in biodiverse ecosystems (Sandbrook et al., 2021).

A key step, and considerable challenge, in reckoning with and addressing the colonial history of and ongoing neocolonial approaches to conservation lies in developing “alternative conceptualisations of trust” (Saif et al., 2022) that reconceptualize “trust building in conservation away from a one‐directional target” (Saif et al., 2022) and toward a true commitment to repair and collaboration with marginalized communities.

### Political dimensions of conservation technology

PAM, and bioacoustics specifically, may be a suitable, cost‐effective method with the potential to fill data gaps in tropical regions, particularly in Africa, where biodiversity data are limited (Becker et al., 2022). Yet, there is a “paucity of Africa‐based bioacoustics literature,” and the integration of the local ecological knowledge (LEK) of rural African communities remains largely understudied (Appiah, 2020). For bioacoustics projects focusing on bird monitoring, this lack of engagement emerges from the colonial and discriminatory past of ornithology, which was marked by “racism and intellectual arrogance among white people” concerning African populations (Jacobs, 2006) despite the technical support delivered by local communities. These colonial patterns extend to current data collection practices. Indigenous and local communities are “seldom…involved in the technology design of conservation tools, which results in the design of non‐intuitive systems” (Muashekele et al., 2019). Critical engagement with the ways PAM can compound and widen the disconnect between formal conservation and communities is necessary to avoid weakening connections to essential local knowledge or resisting colonial and capitalist approaches to participation and conservation innovation (Lorimer, 2015; Ritts & Bakker, 2021).

Scholarship on data justice (DJ) for biodiversity conservation is growing and attempts to respond to these concerns about conservation technology more widely. Building on the established DJ (Taylor, 2017) and environmental data justice (EDJ) (Vera et al., 2019) literature, the concept of conservation data justice (CDJ) was introduced by Pritchard et al. (2022), who acknowledge that neither conservation nor technology is neutral; both are power laden (Robinson et al., 2023; Wickberg et al., 2024). With a focus on PAM, Ritts et al. (2016) reported on Indigenous‐led science collaborations with the Gitga'at First Nation; argue that ecological knowledge production needs to be reoriented as praxis based; and demonstrate how this approach benefits resource management as well as academic and Indigenous community interests.

Sociology, political ecology, and science and technology studies (STS) have long presented clear connections between the political and economic contexts and levels of trust in colonial or postcolonial settings (Gabrys, 2020; Horowitz, 2010; Longdon et al., 2024; Nost & Goldstein, 2022; Pritchard et al., 2022; Skarlatidou et al., 2024). However, deeper interdisciplinary dialogue on, and methodological development for, building trust in projects engaging marginalized communities and conservation technologies is needed to “challenge inequalities…and work towards more pluralistic engagements” (Gabrys, 2020). Without this work, the effectiveness of PM is undermined. Such effectiveness would ideally be coconstructed by scientists and communities to identify how further knowledge about forest environments can enhance livelihoods and conservation research and practice.

We addressed these challenges by documenting our participatory acoustic monitoring engagements with a forest fringe community in Ghana and highlighting the risks, tensions, and opportunities that arose when PAM sensors were introduced in forest community settings. We devised recommendations for building strong and engaged collaborations with participants that attend to the political and justice implications of conservation technologies through 3 key phases: open interrogation, addressing agency, and demonstrating data practices.

### Research team, participants, and positionality

Our study brings together authors from Ghana and the United Kingdom and was informed and guided by our diverse sets of perspectives. The coauthors work in and have perspectives across computer science, human–computer interaction (HCI), European‐centered ecology and ecoacoustics, CS, sociology, and Ghanaian‐centered forestry, forest‐based livelihoods, and natural resource planning. J.L. is an early‐career researcher with familial connections in Ghana, which is her country of heritage maternally and fraternally. J.L. has had the privilege of living and visiting the country several times during her childhood. Before this research, she had not conducted fieldwork in the forested regions of Ghana. J.L. does not conflate her identity as a Black woman with the lived experience of the forest community she works with. This work was conducted in collaboration with research assistants from the Kwame Nkrumah University of Science and Technology (KNUST), Enock Ba, Joseph Kankam, Abena Fosuaa Acheampong, and Priscilla Osei. The participating community had an approximate population of 200 people, mostly farmers dependent on agriculture as their main source of income who specialized in cocoa, cassava, plantain, cocoyam, and, less frequently, maize. There are wider discussions across disciplines about the use of the word *community*; in the Ghanaian context, referring to community members as *villagers* or to their home as a *village* can be interpreted as derogatory. Thus, *community* is a crucial term for Ghanaian participants to use to describe their relations to place. Across the field seasons, we engaged with approximately 70 people in the wider community through community meetings, workshops, and semistructured interviews. For the engagements detailed in this article, *community* refers to a group of 12 individuals: Samuel Teye, Kwadwo Appiah, Yohanne Quadoe, Abena Dufie, Isaka Dramani, Comfort Arkoh, Diana Acquah, Yaa Achiamaa, Ama Kohadu, Agnes Yaa Nkonsah, Akwasi Sarfo, and Kwaku Amoah. Their names are included here to honor their participation and to acknowledge and celebrate publicly their contributions.

## METHODS

We developed our methods in line with, not outside of, community needs and feedback. Although it is uncommon in traditional PAM research, we detailed the social context of our research to highlight the inherent interplay between participatory conservation research and its sociocultural context. Our research design was approved by the Research Ethics Committee for Computer Science and Technology at the University of Cambridge (reference number 1892).

### Study region

Fieldwork was conducted from March to June 2022 in a community on the fringe of the Bosomtwe Range Forest Reserve (BRFR) in the Ashanti Region of Ghana. The BRFR Key Biodiversity Area (KBA) and Important Bird Area (IBA), as defined by BirdLife International, cover 79 km^2^ and are located in the moist semideciduous forest zone. The participating community had an approximate population of 200 people, mostly farmers dependent on agriculture as their main source of income who specialized in cocoa, cassava, plantain, cocoyam, and, less frequently, maize.

### Community entry protocol

It is essential in a participatory or community‐based project to acknowledge, respect, and observe the community entry protocol (Appiah, 2020). Community entry is an essential and important part of building trust with communities in Ghana because it creates a space for mutual dialogue and questioning before research can commence. It shows respect to the elders of the community and gives agency to the community on how, if they do at all, to proceed with collaboration.

Once formal acceptance of the research was granted by the chief, we conducted our first community meeting. J.L. presented the work on a large poster on which the ecological, sociological, and technical motivations of the work were outlined. The motivations and points of interest communicated through the poster emanated from a dominant Western perspective on the importance of forests, the importance of monitoring wildlife, how wildlife can be monitored with acoustic technology, and the importance of community participation in the work. After the presentation of the poster, community members asked questions and shared their ideas about the project. Finally, we passed around a form on which community members interested in being interviewed could provide their name, age, and contact number if applicable.

### Semistructured interviews

After formal collective consent was given, we began the semistructured interviews. Semistructured interviews are a qualitative research method in which “the researcher asks informants a series of predetermined but open‐ended questions” (Given, 2008). The interview questions were compiled before J.L. entered Ghana and were chosen to investigate 4 main research themes: forest perceptions and interactions; wildlife perceptions and knowledge; attitudes on technology and the acoustic sensor; and community needs and interests regarding forest monitoring (question in the Supporting Information Appendix).

Out of the 67 volunteers who put their names forward to be interviewed, we employed stratified purposive sampling to select 15 community members across different ages and genders to ensure a diverse range of perspectives. This method divided the target population into subgroups (strata) and resulted in a random sample from each stratum. The goal for engagement was not to create broad generalizations but to “develop an in‐depth exploration of a central phenomenon” (Creswell & Creswell, 2017). This was accompanied by snowball sampling in which participants suggested members in the community who were knowledgeable about the forest. We conducted 2 test interviews (1 man and 1 woman) to refine the interview process and then spoke with the remainder of the participants for 40–90 min about their knowledge of local wildlife, forest health, and views on ecoacoustic technology. All interviews but one were audio‐recorded.

Following the interviews, we analyzed the data with qualitative coding and thematic analysis, the process by which notes and transcripts from field research are “gradually converted into usable data through the identification of themes” (Austin et al., 2014). We segmented, categorized, and summarized data and clustered related and relevant observations and participant contributions. With the resulting themes, we sought to “build a complex, holistic picture” of the beliefs, motivations, experiences, and perspectives (Castleberry & Nolen, 2018) of participants and the research situation. Many codes were assigned at the beginning of the process, but in each consecutive revision, codes were merged into larger themes that encapsulated the key messages from the transcript data that were generalizable over the different responses collected. The themes that emerged were questioning, agency, proof, and knowledge.

### Participatory workshops

Two workshops were created in direct response to the themes identified from the interviews. These workshops were not planned prior to the fieldwork. Instead, between each interview or workshop, we responded to direct feedback from community members week by week. In this way, the workshops were not accumulations of activities based on what we thought the community would want to engage with but on what they requested themselves.

The first workshop focused on the tangibility of acoustic recording hardware, role‐playing data collection, and building an understanding of the acoustic data itself. This first workshop was attended by 16 villagers (10 men and 6 women) and comprised 4 main activities: deconstruction and reconstruction of an acoustic sensor, collective sensor deployment, bird call quiz, and visualization of collected environmental recordings.

The second workshop followed a short bioacoustic pilot study with 2 acoustic sensors. The participatory activities were explored through 4 key sections of the workshop, each addressing the 4 major data analysis phases commonly used in bioacoustics: visualization, segmentation, pattern matching, and classification. Instead of demonstrating each stage with a complex computational process, we used a low‐fidelity, human‐centered approach using Microsoft PowerPoint and human interaction to make the themes and concepts explored more accessible and to reduce hierarchies based on skill level.

## RESULTS

### Semistructured interviews

Through the initial 6 interviews, it became clear that many community members were suspicious of the research and questioned its purpose, consequences, and the agenda of J.L. Despite receiving a long and detailed description of consent, freedom to refuse recording, and freedom to leave the interview at any time before each interview, to which all but one participant gave consent, we continued to receive questions, expressions of dissatisfaction, and, in particular, concern that the interviews were like interrogations or were part of a larger investigation. This mistrust led concerned community members to warn others not to participate or to give abrupt, quiet answers during interviews.

There followed a period of intense resistance to the research and growing tension, withdrawal, and, at times, aggression toward researchers during field visits. The resistance we experienced was likely a result of the dispositional distrust based on personal histories and existing tensions community members felt with researchers and forest officials who represent institutions of power, extraction, and colonialism. Community members shared that their only previous experience with conservation research involved the arrival of ecologists looking for a rare species. After their investigations, the researchers presented images of the target species and told community members not to visit their nesting site. Regarding state forest officials, there are ongoing reports of mistrust toward and feelings of devaluation by government forest officials who, despite national policy encouraging more collaborative forestry since the late 1980s (Asumang‐Yeboah et al., 2022; Hansen, 2023), seldom involve community members or adopt traditional approaches to forest management (Akamani et al., 2015; Anning & Grant, 2008; Asante et al., 2017; Kumeh, 2023; Hansen, 2023). We addressed the dispositional distrust that positioned us as part of the wider set of extractive colonial institutions, which, given J.L.’s positionality as a Global North researcher at one such institution entering the forest as a scientist, was not unfounded.

In response, J.L. edited and rewrote the interview questions (Supporting Information Appendix). A second community meeting was convened by E.A. X., who built rapport and deepened trust with the community. The community was given the opportunity to change or end the engagement but approved the continuance of the project with the updated questions.

As the interviews progressed, it was clear that we were reaching saturation on many of the questions, at which point we closed off the interview phase. Analysis of the interview responses revealed our key themes: questioning, agency, proof, and knowledge.

### Questioning

The introduction and planned deployment of conservation monitoring technologies evoked questions primarily related to surveillance, law enforcement, and privacy. Responses included:

So, if we are going to mount the machine and we are conversing, will it be recorded or you have to set it when we get there so the conversation will not be recorded?

What about if you left your device here for us to continue we will have issues with the forest guards for entering the forest. What if a member of a community is going to service the device and he is a hunter, sees an animal and kills it or harvest NTFPs [nontimber forest products] will he be in trouble with the forest guard?

When you arrived, people were speculating that you are here to monitor the illegalities happening in the forest, even with the interviews too.

### Agency

Community members advocated for their own operation of conservation monitoring technologies rather than that of external actors. Responses included:

We prefer the community people to take over.

We will be glad if we are taught how to do it and we will do it ourselves.

I would prefer if in the long run the project and monitoring was helped purely by the community to manage themselves.

### Proof

Community members required hands‐on experience with the technologies proposed for deployment and the associated data processing techniques to address their suspicions and better understand the function and purpose of the technology. Responses included:

I want to know the results and listen to the sounds.

If you are able to demonstrate or show us something about the project that is contrary to what the community is speculating, that can even help to make the project successful. And even if and when you are not around, we will continue to make the project a success.

### Knowledge

Conservation technology was perceived as an exciting opportunity for community members to gain knowledge, both technical and related to community conservation agendas. Responses included:

Yes, it is very important. In this world, you have to learn and gain knowledge so it [the AudioMoth] is good. It is a form of education.

It will help us to know the total number, the different species and their relative abundance.

### Dynamic consent through participatory workshops

Reflecting on the success of the slower, less formal, more relational, and participatory community meeting format, it became clear that community members required an interactive process that responded to their concerns, apprehension, curiosity, and agency. A pilot deployment of ecoacoustic sensors was planned to follow the semistructured interviews, but a responsive, pace‐sensitive, and dynamic consent process was adopted to attend to and challenge the prevalent power dynamics, center community agency, and prioritize trust and relationship building before beginning monitoring in earnest. This dynamic consent process emerged as a set of participatory and interactive workshops, detailed below, that ensured community members could make an informed decision on their long‐term involvement in the research and acknowledged the fact that consent and trust are not fixed, but fluid, and influenced and informed by the “reciprocal perceptions of, and interactions between” researchers and participants (Horowitz, 2010) and are “social construction[s] of ongoing mutual satisfaction that can be broken if one party ceases to respect their obligations” (Moustard et al., 2021).

Given the discomfort communicated by participants in the semistructured interview phase, observations from the workshops were not recorded; instead, multiple sets of written notes were made to ensure that community members felt uninhibited in their engagement.

### Making ecoacoustic monitoring tangible

**FIGURE 1:**
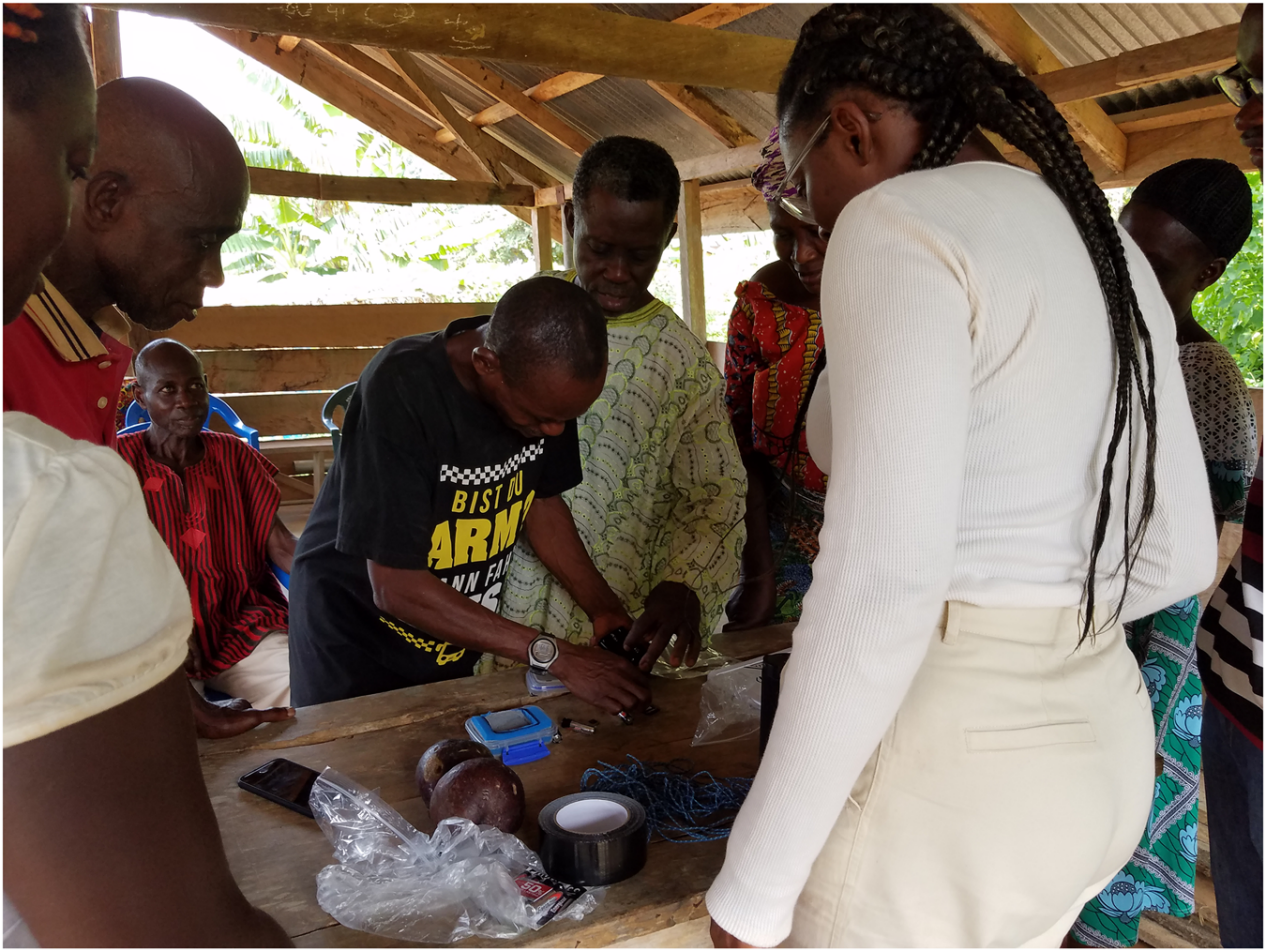
Community members and J.L. engaging in a participatory workshop in which AudioMoth acoustic sensors were constructed and deconstructed.

In the first activity, small groups of community members explored and inspected the decomposed components of the AudioMoth acoustic sensors (e.g., taking out and inspecting different elements of the sensor from the SD card and batteries to the foam padding in the protective casing we configured to reduce costs), and when they were inclined, they put the sensor back together (Figure 1). This activity responded to the themes of questioning and proof and provided community members the space to freely inspect and ask questions about the device that would be embedded in their forest and the opportunity to experience and acknowledge small technical considerations (e.g., making sure the case hole did not cover the microphone of the AudioMoth).

Community members felt uninformed about the process of deploying the sensors. There were concerns about how they would or should engage with the forest when the sensors started recording, and whether they would be recorded in an area of the forest without their knowledge. To facilitate an understanding of how the sensors would be deployed, responding to the themes of questioning, agency, and proof, community members led discussions and made decisions on the trails where the acoustic sensor would sit for a short period (15–30 min). Once a suitable location was found, somewhere close to the village for accessibility but not too close to people's houses, we walked as a group to set up the sensor, led by one of the key hunters in the village. This activity gave community members agency in the experimental design and facilitated leadership in sensor deployment and better appreciation of its logistics, such as proper placement and camouflaging techniques for camouflage.

Community members initially mistrusted our explanations that we could identify wildlife using sound, believing it was a dishonest cover for image‐based surveillance. To build trust and center their knowledge, we returned to the small wooden church—our regular meeting point—to hold a bird‐call listening and identification session. This session served 2 purposes: first, it demonstrated how ecoacoustic methods work without cameras; second, by listening to and identifying bird calls together, community members engaged in enthusiastic discussions, providing an opportunity for knowledge exchange, during which they told stories about and shared their experiences with certain species that lived in the forest.

After the listening and identification session, we sought to address community members’ suspicions around the ability to isolate species calls and human voices from a sound wave. To provide proof of ecoacoustic data processes, we conducted a live demonstration of retrieving the data from the sensor and using the Audacity (Audacity, 2017) sound editor to visualize the recording as both a waveform and a spectrogram. Community members were then able to inspect different segments of the recording and ask questions about the different ways of visualizing sound. By visualizing the waveform and the spectrogram side by side, community members were able to see the content of ecoacoustic data and became animated by the truth in what we had described and also by the variety of patterns in the spectrogram representing different sources of sound from the forest

### Human‐centered ecoacoustic data

The second workshop responded to feedback from community members that there would be more trust if they were able to interact more deeply with the data collected in their forest. It followed a more formal bioacoustic pilot study in which 2 acoustic sensors were deployed with 4 community members (3 hunters and 1 farmer) for 1 week. Agency was given to the community field team to choose the sensor locations and ensure that the sensors were deployed in areas of perceived high biodiversity that did not intersect with heavy human traffic. This deployment was understood not as the beginning of the formal deployment but as a trial that would be used to inform the community on whether they consented to the full scope of work being proposed. Here, the bioacoustic pilot, alongside the analysis activities, served as part of the process of providing proof of the experimental and recording process. It demonstrated not only the type of data being collected but also the techniques used to analyze it.

In the visualization part of the workshop, side‐by‐side images of a waveform and spectrogram of the same recording were shown, and the community responded to these images by discussing what they saw (e.g., if they thought one visualization was more instructive than another or how each visualization connected them to the sound). This was situated as a recap of the last activity in the first workshop and allowed community members to narrate what they were seeing in the spectrograms and to observe how the calls played to them appeared in the spectrogram itself.

In the segmentation activity, an example recording with no visualization was played with the aim of counting how many distinct vocalizations there were. Community members listened to the recording as many times as needed, guessing after each time how many distinct species they could hear. The listening was followed by an inspection of the associated spectrogram of the same recording and a discussion on whether community members’ guesses had changed, the difference in information obtained by human listening alone in contrast with visualization, and how easy or hard it was to differentiate sounds by ear and sight.

In the pattern matching activity, community members were shown 4 spectrograms from 2 distinct vocalizations and asked to match them. Two of these spectrograms were identical, and the other 2 were different spectrograms of the same species call. Although community members easily matched the 2 identical copies, they debated whether the 2 different spectrograms of the same species could be counted as a pair, given they were not exactly the same. This led to a discussion about how machine learning classifies species and the error boundaries algorithms use to determine whether spectrograms belong to the same category.

In the classification activity, community members were played example recordings and asked to classify the vocalizations they recognized. To decenter solely Western conceptions of classification through naming and to probe whether there existed differences in the ways community members classified wildlife species, we did not just ask for classifications by name. Instead, members were given a list of other identifying indicators, such as color, size, time of activity, and importance to the community.

### Community feedback and reflections

The community engagements concluded with an open feedback session from community members, where they were given the opportunity to reflect on the time they had spent engaging with the research. Reflecting on the pause taken to edit and rewrite the semistructured interviews, community members likened the meeting convened by E.A. to an event in the forest where,

the alpha male [monkey] does a hum for like 3 times at distanced intervals before it does kia kum for 3 times…kia kum is heard from the leaders…this proverb is described by that kia kum made by the monkeys as it is the Alpha male who leads and who guides and advocates. This prevents doubt.

In contrast to the periods of tension and resistance that were prevalent at the beginning of the field trip, community members communicated that the opportunity to more deeply engage with the research through the participatory workshops allowed them not only to feel more trusting but also to cultivate feelings of intrigue, interest, and excitement in the project. One participant said,

If it was not for this device, I wouldn't have known that some of these species exist. So, I am intrigued with the device and will commend the idea.

Another participant said,

I want to commend you on the fact that when you came here you said that you were here for the animals and truly, you have just recorded the animals. This has built trust between you and us.

On trust again, another participant shared that,

Through you, we have become more open to the researchers, so when you go, tell other researchers that [we] are open to research.

## DISCUSSION

Our results showed that an unhurried, dynamic, and extended approach to consent is necessary for trust building in participatory biodiversity monitoring projects. Although consent is often seen as a procedural task (Pham et al., 2015) or obstacle (Merino, 2018), in participatory research—often marked by the signing of a form or a verbal response to a consent request—our findings revealed that consent must be an ongoing process that extends through all stages of the research. Similar to the concept of dynamic consent introduced in this work, Newing et al. (2024) highlight the need for “on‐going FPIC” and provide “a framework within which changes can be made, periodic reviews of the agreement conducted, and community consent maintained throughout the life of a conservation activity… [and] is inherent to the on‐going nature of human rights due diligence.”

Rather than a hindrance or inconvenience to the research process, we found this approach to be generative in that it opened up new avenues for ecological enquiry and research and was responsive to the challenges inherent in PM and design research (Gabrys, 2022; Shilton & Estrin, 2012; Shilton et al., 2008). A dynamic consent process resists the “competitive timelines, budgetary constraints, and specialized disciplinary interests” (Ritts et al., 2016) that often make it difficult for researchers to build trust and forge strong relationships with community partners (Adams et al., 2014; Jalbert, 2011; Pham et al., 2015; Ritts et al., 2016). This requires reconciling uneven power dynamics in which researchers are often seen to “driv[e] the pace [and] thus ultimately control” (Adams et al., 2014) the research process, which can often lead to communities feeling “compelled to consent because they perceive researchers (who may also be clinicians or nongovernmental organization representatives) as authority figures and/or potential sources of assistance” (Brear, 2018).

Based on our findings and observations, we devised 3 practical suggestions for researchers, practitioners, and resource managers to move from concern to consent when engaging Indigenous Peoples or local communities in participatory conservation research. Although the following recommendations emanate from participatory research conducted in a specific context with a specific community, their implications for conservation research and practice can be relevant beyond the Global South–Global North divide and extend to communities living closely in a variety of ecosystems and engaging with a wide spectrum of conservation technologies. In the following sections, we use the term *community members* to encompass any nonacademic participant in the research (e.g., farmers, natural historians, or even in‐country researchers who may be less familiar with conservation technologies).

### Respond to suspicion

To prioritize the concerns of community members and challenge the extractive nature of participatory research, researchers must become comfortable with being interrogated. The introduction of technology in an environment where exposure to technology is low can raise suspicion and concern. There may be suspicion of the researcher's intentions, allegiances, and agendas, as well as of the technology being used itself. This suspicion and discomfort may not always be verbally communicated and could be easily missed if a researcher is not attuned to cultural communication styles; it is common in African cultures that displeasure or disagreement is signaled through quietness or silence (Bidwell, 2016). The setting of boundaries by community members on what they were comfortable or uncomfortable speaking about aligned with observations that “refusal, and stances of refusal in research…[mark] what is off limits, what is not up for grabs or discussion, what is sacred, and what can't be known” (Tuck & Yang, 2014). Researchers must be prepared to be questioned before questioning and to build in activities that move the dynamic from researcher as enquirer to a mutual pursuit of understanding.

Conservation hardware introduces and prompts questioning and the interrogation of power and agency in participatory research. Many objects that researchers assume to be neutral or trivial (e.g., sensors, SD cards, sensor cases, scientific equipment) can be threatening or concerning to community members, especially when they have not previously interacted with such devices. Without providing community members the ability to interrogate the technological devices used to monitor biodiversity so they can explore and understand their technical capabilities and limits, many questions about how those tools might threaten community members’ lives or activities go unanswered, and the status quo of the researcher as leader and technical superior goes unaddressed. This power dynamic can lead community members to feel either that the researcher is hiding the true intent or function of the device or that they are not skilled or trusted enough to engage with it. Scientific equipment should be seen and used as probes to start conversations and prompt questioning in the consent process. Positioning these objects as points of inquiry allows community members to feel involved in and to remove ambiguity around the research process while attending to the power dynamics that arise when a scientist is very familiar with their own equipment, whereas community members are kept in the dark. We also observed how this approach leads to more successful collection of data as community members become familiar with equipment, gain the skills to troubleshoot problems, and continue data collection without the researcher being present, an essential part of project longevity.

### Address agency and center community knowledge

We found that opportunities for community agency and leadership within the consent or exploratory phases of PM projects allowed local knowledge to be central. The facilitation of self‐direction in the consent process models the level of participation that will continue through later phases of the research cycle and communicates the level of respect and priority given to the knowledge and experiences held by community members. Opportunities for community leadership range from decision‐making, such as the planning of an experimental pilot, to the ability to set the timing, location, pace, and direction of meetings.

The ways in which community members are enabled to lead the research or exercise their agency cannot be prescribed; it must instead be given the space to emerge when deemed necessary or requested. This is true not only in instances where community agency is limited by the research design to a specific phase, such as data collection, but also when it is expected at every stage of the research. There were clear moments when the agency of community members was exercised by the demand for the researcher to prove their practices or to prove their knowledge, rather than information being solicited by the community members. We found that community members responded positively and that trust was built when their needs were responded to in real time and when the research process was shifted by the researcher, demonstrating their reflexivity, attentiveness, and attunement to those needs.

It is important to create space for community members to share their local and cultural knowledge outside of research activities or agendas alone. Community members must feel that there is a genuine interest in their knowledge, even if it is not immediately exploitable by the researcher. This knowledge, often presented through storytelling, may not necessarily be related to the overarching research goals, but genuine interaction with these stories is an important part of reassuring the community that genuine relationship building, not just knowledge extraction, is occurring. In African contexts (as well as other cultures), we suggest that offers of knowledge exchange occur over local or popular food or drinks, providing participants with gratitude for the time they give and engaging in the cultural practice of storytelling and relationship building that occurs over and through food.

### Demonstrate data practices

The phases of the research that focused on the tangible and practical elements of ecoacoustic research, sensor deployment, and data analyses made the largest impact on the dynamic between researchers and community members. A recurring concern from participants involved the data collection strategies, the experimental design, and the need for the researchers to prove the practices and methods they claimed they would use.

We found that role‐play and trials addressed these concerns by allowing community members to lead the research and providing the necessary training and education for them to do so within the project and beyond their relationship with the researcher. This is important given the ongoing issues with longevity seen in conservation projects in which community members are engaged for the duration of the project's funding, after which point, they are unable to continue the research or receive any associated benefits.

Researchers must approach these pilots and trials not as the beginning of formal data collection but as part of the consent process. It is through these activities that community members discuss and decide how they feel about the proposed work, what limits they wish to set, and what amendments to propose to better fit their needs. Researchers should prepare hands‐on sessions that break down their research methods and present these sessions to community members prior to formal data collection. These sessions should have a planned and accessible structure and provide time for unguided, undirected exploration. These pilot sessions allow community members to be well informed about the research they are agreeing to and to take ownership and leadership of the research process and provide a more dialogic approach to PM in which community members have genuine power to understand, engage in, and inform much of the research approach.

Participation can be a constant negotiation of trust and agency, and to move from concern to consent, conservation technology researchers and practitioners must engage in this negotiation through the 3 key research phases we discussed, which we conceptualized as part of a dynamic consent process. Engaging in these 3 phases shifts PM engagements from sources of extraction to more dialogic, adaptable, and responsive approaches to research that build agency, result in a cocreated research process, and lead to more trusting exchanges with community members. Such methods can ensure that tools, such as PAM, are used in ways that further ethical and just conservation practices and outcomes.

## Supporting information



Supporting Information
