# Supplementary material for: Building trust with marginalized communities in participatory acoustic monitoring through dynamic consent
Source: Conserv Biol. 2026 Mar 9;40(2):e70222. doi: 10.1111/cobi.70222 (PMC13036284; doi:10.1111/cobi.70222)
Supplement: Supplementary file 1 — Supporting Information [file COBI-40-e70222-s001.pdf]

Supplementary Information

Interview Schedule [Pre-Field Work]

The objective of these interviews is to form a foundation for community relationships and understand the context of forest conservation or engagement in the chosen community. This interview will provide time for me, as the researcher, to listen to community members and relationship build, rather than to extract specific information. This interview will occur prior to the introductory presentation where I will present who I am and what my work is.

Opening - to build rapport and make the interviewee feel comfortable

Introduction: Hello there, my name is ___________ and I am here in Ghana undertaking my doctoral research. Here is _____ my field collaborator (field assistant) who will be translating for me.

Gratitude: Thank you very much for giving up some of your time to talk with me today, I really appreciate it and am looking forward to our conversation

Study Purpose: My research is focused on making sure that technologies we use to conserve and monitor the forest, are not only built and informed by scientists but communities too. The aim is to undertake a monitoring project, using audio sensors, to monitor the biodiversity of wildlife in Ghanaian forests in a way that is culturally informed and useful for the community. The purpose of this interview is to get a better understanding of your connection with the forest, how you engage with it and its importance to you.

Interview Details: With your permission, I will be audio taping this session so that I don't miss anything important you say. I will also be taking some hand written notes as you speak. The Interview should last between 40 and 60. As noted in the information sheet, your responses will be kept anonymous unless you want to be identified and you can decide whether or not you want to be recorded on the audio tape. Any information shared in the next hour or so will be kept safe on my computer in an encrypted file and backed up to a folder that cannot be read by anyone but me.

Consent: Do you consent to taking part in this interview and having your responses recorded?

Topic 1 - Personal Background and Forest Engagement

1. How long have you lived in this region?
    a. Are you originally from this region?
2. How often do you engage with the forest?
    a. What encourages or demands of you to enter and engage with the forest?
3. Could you describe a typical day when you engage with the forest?
4. When you are in the forest, what is the main thing you notice or see?
5. How often do you listen to the forest and wildlife sounds?

Topic 2 - Forest Observations

1. Does the forest seem healthy to you?
2. How has the forest changed over your time here?
3. Do you notice less or more wildlife in the forest?
4. Do you feel that the forest is abundant?
5. Do you think anything is affecting the forest negatively?

Topic 3 - Forest Values, Traditions and Importance

1. Is the forest important to you?
    a. If so, why? If not, why not?
2. How do you feel when you enter the forest?
3. How connected do you feel to the forest? Do you feel like you know it well?
4. What did you learn about the forest when you were growing up?
5. Do you keep any traditional or spiritual practice with the forest?
6. Do you know how your ancestors used this forest?
    a. Do you use it in the same way?

Topic 4 - Conservation

1. Is there a species of animal that is important in this region?
    a. Why is it important?
    b. What does it sound like?
    c. What does it look like?
    d. Where does it usually live?
    e. Do you see this species often?
2. Have there been actions to conserve the forest from the community?
3. Do you feel that the forest is well conserved?
4. Are there any pressures that the forest is under?

Topic 5 - Response to Tech

1. Have you used technology to conserve or monitor the forest before?
2. What did you first think when you heard about the audio sensors?
3. Do you think the sensors would be useful?
    a. How?
4. Do you have any concerns about this technology?
5. What would you find more important, understanding the general health of the forest or monitoring a specific species of animal?
    a. Why?

Updated Interview Schedule [During Field Work]

Part 1 - General Information

1. Are you originally from this region?
    a. If not, how long have you lived in this region and where were you originally from

    b. if so, how many generations of your family are from this village?
2. What do you do for your livelihood?

Part 2 - Forest Values and Interaction

1. Is the forest important to you?
2. Is wildlife important to you?
    a. If so, why? If not, why not?
3. Do you think it is important to monitor and conserve wildlife?
    a. why?
4. Are there any spiritual or traditional rituals or practices that you take in the forest?
5. Are there people in the community who are passionate or knowledgeable about wildlife ?

Part 3 - Biodiversity and Wildlife Abundance Observations

1. Do you know what biodiversity is?
    a. If not, explain
    b. If yes, ask for explanation
2. Does the forest seem biodiverse to you?
    a. Are the areas of the forest that are more diverse with animals than others?
3. Has the animal diversity changed over your time here?
    a. If so, how?
4. Do you think anything is affecting the animal diversity negatively or making it less abundant?
    a. if so, how does this make you feel?

Part 4a - Wildlife Knowledge (Open - this set of questions allows any other species to come up I may be unaware of)

1. How often do you listen to the forest and wildlife sounds?
2. Are there species of animal that you hear or see often?
    a. Can you describe what it looks like?

    b. Can you describe what it sounds like?

    c. What do you call it?

    d. How often do you hear/see it?

        i. every day
        ii. once in 14 days
        iii. once in30 days
        iv. once in 1–3 months
        v. once in 3–6 months
        vi. once in a year

e. Where does it roost/nest or do you usually see it?

f. Do you think it is abundant? (Likert scale, see: https://besjournals.onlinelibrary.wiley.com/doi/10.1111/2041-210X.13773)

   i. 0 (when the species was ‘absent’),
   ii. 1 (low abundance),
   iii. 2 (medium abundance)
   iv. 3 (high abundance; Van Holt et al., **2010** , **2016**).

g. ii. Do you think it is becoming more or less abundant?

h. Do you think it is important?

3. Have there been actions to conserve or monitor this (these) animal(s) in your community?
   a. If so, what?

Part 4b - Wildlife Knowledge

Show an image of each species targeted.

1. Can you describe what it sounds like?
2. What do you call it?
3. How often do you hear/see it?
   a. every day
   b. once in 14 days
   c. once in30 days
   d. once in 1–3 months
   e. once in 3–6 months
   f. once in a year
4. Where does it roost/nest or do you usually see it?

5. Do you think it is abundant? (Likert scale, see: https://besjournals.onlinelibrary.wiley.com/doi/10.1111/2041-210X.13773)

   a. 0 (when the species was ‘absent’),
   b. 1 (low abundance),
   c. 2 (medium abundance)
   d. 3 (high abundance; Van Holt et al., 2010 , 2016).

6. ii. Do you think it is becoming more or less abundant?

7. Do you think it is important?

   a. What is it called in the local language?

Part 5 - Technology Perspectives

1. Have you seen technology or phones used to conserve or monitor the forest before?
2. What was your first impression when you heard about the audio sensors? (show again)
    a. have you seen something like it before used in the forest?
3. Do you feel you understand how the sensor works?
    a. if not, explain
4. Do you own a mobile phone?
    a. What make is it?
    b. What do you use it for?
5. Does your phone have external memory, like an SD card?
6. How much do you use your phone?
7. Do you use any other technological device?
8. What would make using technology in this project more useful and accessible to you?
    a. How?
9. Do you think technology can give you power?
    a. how?
10. What kind of data or results would you be interested in learning about wildlife?
11. How would you use the data and results you receive in your community?
12. What would be the best way for the data and results to be communicated?
13. What part of the research is most interesting to you? The technology or finding out more about the species?
14. Do you have any concerns about this technology?

Part 6 - Benefit Sharing

1. What benefits do you think this research will bring to the community?
2. Do you think the research will positively impact your life?
3. If the project was successful, what would that look like?
